# Dental professionals' use of personal protective equipment during COVID-19: a cross-sectional study in China

**DOI:** 10.3389/fpubh.2023.1183580

**Published:** 2023-07-03

**Authors:** Qinjie Wan, Lei Han, Xin Yang, Shaoling Yu, Xiaohong Zheng

**Affiliations:** ^1^School of Energy and Environment, Southeast University, Nanjing, Jiangsu, China; ^2^Department of Orthodontics, Nanjing Stomatological Hospital, Medical School of Nanjing University, Nanjing, Jiangsu, China; ^3^Department of Pediatric Dentistry, Nanjing Stomatological Hospital, Medical School of Nanjing University, Nanjing, Jiangsu, China

**Keywords:** COVID-19, dental professionals, personal protective equipment, infection control, survey

## Abstract

**Background:**

Appropriate use of personal protective equipment (PPE) could significantly reduce the risk of viral transmission and infection. This study aimed to assess the use of PPE among dentists during the COVID-19 pandemic in China, explore its influencing factors, and provide some practical recommendations.

**Methods:**

An online cross-sectional survey was conducted among 384 Chinese dentists in September 2022. The questionnaire comprised a series of questions about demographic characteristics, compliance with proper PPE use, personal barriers to use, and exposure risk estimation.

**Results:**

Of the 384 respondents, 57.3% had unacceptable compliance with the proper use of PPE during COVID-19. Medical surgical mask is the most common for dental professionals to wear (93.8%), followed by goggles or face shield (63.8%), and isolation gown (53.1%). Unexpectedly, only 63.3% of respondents always change masks with guidelines. The condition for changing goggles/face shields and isolation gowns is even worse (45.6 and 37.0%, respectively). Visual barriers, physical discomfort, complex procedures, and heavy workload were the most common personal barriers to use. According to the results of Chi-square test and correlation analysis, PPE use compliance was associated with age, years of practice, medical institution type, and exposure risk estimation.

**Conclusion:**

Chinese dental professionals need to improve their compliance with the proper use of PPE, especially those in the 31–40 age group, with 11–15 experience years and working in private dental clinics. Increasing compliance with PPE may be achieved by addressing personal barriers to use, human resource shortages, and perceptions of exposure risk.

## 1. Introduction

The novel coronavirus disease (COVID-19), caused by the severe acute respiratory syndrome coronavirus 2 (SARS-CoV-2), has become the most significant public health emergency worldwide since the first case was publicly reported. According to the World Health Organization (WHO), as of November 27, 2022, there have been over 636 million confirmed cases and over 6.6 million fatalities of COVID-19 globally (1).

Healthcare workers (HCWs) are at the forefront of the fight against the COVID-19 pandemic. The pandemic has had an adverse effect on the global healthcare workforce, including the risk of infection (2). In many countries and regions, it has been reported that a substantial proportion of HCWs are infected (3). This is especially true for dental professionals (4). Dental procedures have the characteristics of close contact between HCWs and patients, lengthy operation time, and routine use of high-speed handpieces, ultrasonic scalers, and other high-speed devices, resulting in a high risk of SARS-CoV-2 infection. In dental care settings, pathogenic microorganisms can be transmitted through a variety of routes, including inhalation of suspended airborne microorganisms (5, 6), direct contact with body fluids like blood and saliva (7, 8), mucous membrane contact with infectious aerosols and droplets generated by infected individuals (9), and indirect contact with contaminated instruments and environmental surfaces (10). The risk of infection for professionals and other patients would greatly increase because of the unawareness of the asymptomatic patients. Therefore, dental professionals are classified as very high-exposure risk jobs by Occupation Safety and Health Administration (OSHA) (11).

Due to the specificity of dental treatment, there are many other pathogens present in the dental environment besides SARS-CoV-2 that can cause airborne contamination with organisms, such as tubercle bacillus and Hepatitis B virus (12, 13). As a result, dental professionals are generally recommended to use personal protective equipment (PPE) to reduce exposure (14), mainly including wearing a mask, goggles or a face shield, and an isolation gown (15, 16). This issue was previously often overlooked, but the outbreak of the COVID-19 pandemic has led to its reemphasis and widespread attention. However, existing surveys and escalating case numbers suggest that these guidelines are not well followed in many healthcare practices globally (17). Poor PPE use practices in dental institutions were also reported by studies in many other countries. A survey conducted in Pakistan showed that only 50% of dentists have compliance with PPE wear (18). An Indian study revealed a dearth of understanding on the proper use of PPE in dental treatment (19). Despite strong evidence of gaps in dental professionals' PPE use, we have limited empirical knowledge of these gaps, as well as the barriers and facilitators of compliance among Chinese dental professionals.

This study aimed to assess the use of PPE among dental professionals during the COVID-19 pandemic in China, explore its influencing factors, and provide some practical recommendations for dental participants.

## 2. Materials and methods

### 2.1. Study design and study participants

We conducted an online cross-sectional survey in September 2022. Dental professionals working in China, regardless of their institution, in either public general hospitals, public stomatological hospitals, private stomatological hospitals, or personal dental clinics, were included in the study. The questionnaires were constructed using Questionnaire Star, a free online survey tool that is extensively used in China. The web link and QR code generated by this platform were made accessible on social media sites linked with dental professional groups in China; participants could click the link or scan the QR code to complete the survey.

### 2.2. Ethics approval and consent to participate

The study followed the principles of the Helsinki Declaration. Ethical approval was granted by the Ethics Committee of Zhongda Hospital of Southeast University (No. 2022ZDSYLL306-P01). There was an informed consent on the front page of the questionnaire, which introduced the background information, the objective of the study, the voluntary nature of participants, and declarations of confidentiality and anonymity. Before beginning to fill out the questionnaire, participants were required to read and sign the informed consent.

### 2.3. Sample size calculation

The sample size was calculated by the Raosoft sample size calculator, an online website built for population surveys. A minimum sample size of 384 dental professionals was calculated by considering the margin of error of 5%, the confidence interval of 95%, and the total of 278,000 licensed dental professionals in China reported by the National Health Commission of China (20). The entrance of the questionnaires was closed as soon as the number of valid questionnaires reached the requirement.

### 2.4. Questionnaire elements and scoring

A self-administered questionnaire was created after a review of protection standards for stomatological hospitals during the epidemic period of COVID-19 (21). Fifteen dentists served as a pilot sample to test the questionnaire's acceptability and clarity, and the responses obtained were not included in the study analysis.

We applied the following settings to assure questionnaire validity: (1) all core questions were mandatory; (2) logical jumps were set; and (3) each Internet Protocol address could only be submitted once. In addition, the returned questionnaires that had obvious logical mistakes or took < 120 s to fill out were eliminated.

The self-administered questionnaire comprised four sections. The first section was demographic characteristics, including gender, age, marital status, education, experience, and type of medical institutions.

The second section consisted of 6 items that assessed the study participants' compliance with the proper use of PPE (surgical masks, goggles or face shields, and isolation gowns), including wearing by requirement and changing on time. “Always,” “sometimes,” and “never”, respectively, received 2, 1, and 0 on the compliance scale, and the full score is 12 points. Participants scoring ≥80% were deemed to have acceptable compliance, while those scoring < 80% were deemed to have unacceptable compliance.

The third section consisted of 6 items that explored personal barriers to the proper use of PPE. The dental professionals who could not always wear by requirement and change on time would give their reasons for their behaviors.

The fourth section consisted of 14 items that inquired about the dentist's exposure risk estimation and corresponding PPE use, toward 14 common dental procedures of three types, including orthodontic treatment, periodontal treatment, and filling or root canal treatment. The participants scored the exposure risk for each dental procedure they have engaged in. From 0 to 5, the higher the score, the higher the exposure risk. According to the use of each PPE (surgical masks, goggles or face shields, and isolation gowns), use is scored as 1 point, and non-use is scored as 0 point, and the PPE use scores of each dental procedure are obtained by adding them up.

### 2.5. Statistical analysis

All statistical analyses were conducted using IBM SPSS Statistics (version 25.0, SPSS Inc., Chicago, USA). Descriptive statistics were reported using means and standard deviations (SD) for quantitative data and frequency with percentages for qualitative data. A Chi-square test with the partition of χ^2^ method was utilized to examine the statistical differences in the proportion of acceptable compliance. A two-tailed Spearman correlation analysis was carried out to examine the relationship between exposure risk scores and PPE use scores. All the assumptions of the statistical methods were met. We considered the data statistically significant when *p* ≤ 0.05 with a confidence level of 95%.

## 3. Results

### 3.1. Participants' characteristics

This study included 384 dental professionals from 20 Chinese provinces, forming a validity rate of about 97.9% (384 valid questionnaires out of 393 returned questionnaires). The participants' characteristics are shown in Table 1. Generally, there are 123 males (32.0%) and 261 females (68.0%). The majority were under 40 years old (65.3%) and had < 15 years of practice (64.1%). The distribution was consistent with national human resource statistics on dental health. A total of 191 (49.7%) dental professionals had bachelor's degrees. The largest number of doctors were employed in private dental clinics, accounting for 31.3%, followed by public general hospitals, private stomatological hospitals, and public stomatological hospitals.

**Table 1:** Demographic characteristics of the study participants (*N* = 384).

**Variable**	**Frequency**	**Percentage (%)**
**Gender**		
Male	123	32.0
Female	261	68.0
**Age (years)**		
≤ 30	90	23.4
31–40	161	41.9
41–50	98	25.5
>50	34	9.1
**Marital status**		
Married	239	62.2
Single/unmarried	145	37.8
**Academic degree**		
Associate degree or below	91	23.7
Bachelor's degree	191	49.7
Master's degree or above	102	26.6
**Years of practice**		
≤ 5	70	18.2
6–10	85	22.1
11–15	91	23.7
16–20	50	13.0
>20	88	22.9
**Type of medical institution**		
Public general hospital	115	29.9
Public stomatological hospital	73	19.0
Private stomatological hospital	76	19.8
Personal dental clinic	120	31.3

### 3.2. Dental professionals' compliance with the proper use of PPE

The mean compliance score was 9.26 ± 2.15, and Cronbach's alpha for its internal consistency was 0.707. Two hundred and twenty (57.3%) participants had unacceptable compliance with the proper use of PPE during the COVID-19 pandemic. The PPE use of 384 dental professionals is shown in Table 2. During nursing, diagnosis, and treatment, the proportion of dental professionals who can always wear a surgical mask, goggles or a face shield, and an isolation gown were 93.8, 63.8, and 53.1%, respectively. The proportions of dental professionals who can always change surgical masks on time (after 4 h of use or immediately if the masks become moist or splattered), change goggles or face shields on time (when they are contaminated with blood, body fluids, or secretions), and change isolation gowns on time (after 4 h use or immediately if the gowns become moist or splattered) were 63.3, 45.6, and 37.0%, respectively.

**Table 2:** Distribution of the responses to PPE use (*N* = 384).

**Variable**	**Response** ***n*** **(%)**		
	**Always**	**Sometimes**	**Never**
**Surgical masks**			
Wear by requirement	360 (93.8)	17 (4.4)	7 (1.8)
Change on time	243 (63.3)	130 (33.9)	11 (2.9)
**Goggles or face shields**			
Wear by requirement	245 (63.8)	130 (33.9)	9 (2.3)
Change on time	175 (45.6)	189 (49.2)	20 (5.2)
**Isolation gowns**			
Wear by requirement	204 (53.1)	162 (42.2)	18 (4.7)
Change on time	142 (37.0)	188 (49.0)	54 (14.0)

### 3.3. Personal barriers to PPE use

**Figure 1:**
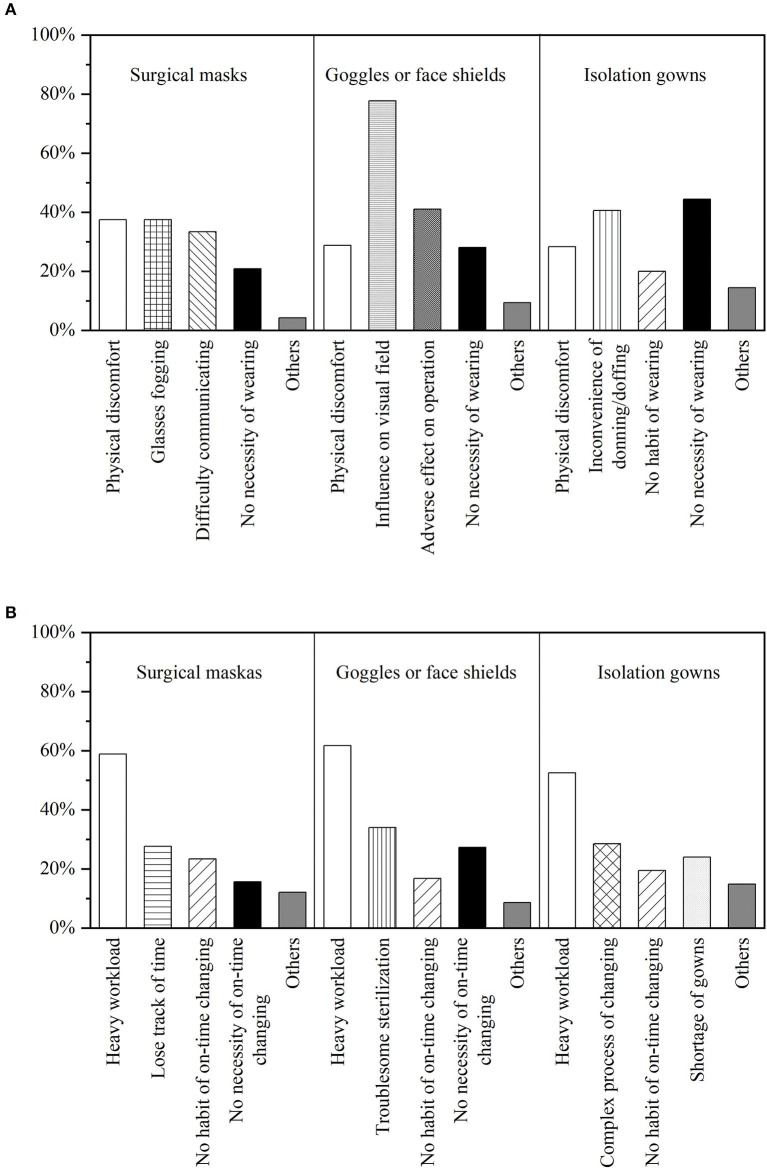
The proportion of personal barriers to wearing by requirement **(A)** and changing on time **(B)**.

We collected and categorized the barriers to the proper use of each type of PPE reported by our participants (Figure 1). Physical discomfort (37.5%) and glasses fogging (37.5%) were the most frequently cited barriers to wearing surgical masks by requirement. A large proportion of dental professionals complained that goggles or a face shield made it more difficult for them to perform duties owing to their influence on visual field (77.7%) and their adverse effect on operation (41.0%). For isolation gowns, 44.4% of dental professionals considered them unnecessary to wear, and 40.6% complained about the inconvenience of donning/doffing. Over half of our participants attribute their poor practice of on-time change to heavy workload, the proportions of surgical masks, goggles or face shields, and isolation gowns were 58.9, 61.7, and 52.5%, respectively. Additionally, the complex and time-consuming procedures of eye protection sterilization (34.0%) and gown changing (28.5%) were reported as important barriers to on-time change.

### 3.4. Relations between compliances with the proper use of PPE and demographic factor

The proportions and statistical significance (χ^2^-test) for each demographic variable by the outcome variable for PPE compliance are presented in Table 3. There were significant statistical differences in age, years of practice, and type of medical institution (all *P* < 0.05), but there were no statistically significant differences in gender, marital status, or education. A lower proportion of acceptable compliance was reported by the dental professionals in the 31–40 age group (32.3%) and the dental professionals with 11–15 years of experience (72.5%). The dental professionals in private dental clinics were less compliant with the proper use of PPE, while those working in public general hospitals had higher compliance.

**Table 3:** Relations between demographic factors and compliance with the proper use of PPE.

**Variable**	**Compliance with the proper use of PPE**		**χ^2^**	**P**
	**Unacceptable** ***n*** **(%)**	**Acceptable** ***n*** **(%)**		
**Gender**				
Male	73 (59.3)	50 (40.7)	0.313	0.576
Female	147 (56.3)	114 (43.7)		
**Age (years)** ^*^				
≤ 30^A^	44 (48.9)	46 (51.1)	12.749	0.005
31–40^B^	109 (67.7)	52 (32.3)		
41–50^AB^	51 (52.0)	47 (48.0)		
>50^A^	16 (45.7)	19 (54.3)		
**Marital status**				
Married	142 (59.4)	97 (40.6)	1.165	0.280
Single/unmarried	78 (53.8)	67 (46.2)		
**Academic degree**				
College and below	50 (54.9)	41 (45.1)	3.127	0.209
Undergraduate	104 (54.5)	87 (45.5)		
Master and above	66 (64.7)	36 (35.3)		
**Years of practice** ^*^				
≤ 5^A^	34 (48.6)	36 (51.4)	20.8	0.002
6–10^AB^	50 (58.8)	35 (41.2)		
11–15^B^	66 (72.5)	25 (27.5)		
16–20^AB^	31 (62.0)	19 (38.0)		
>20^A^	39 (44.3)	49 (55.7)		
**Type of medical institution** ^*^				
Public general hospital^A^	56 (48.7)	59 (51.3)	8.723	0.033
Public stomatological hospital^AB^	44 (60.3)	29 (39.7)		
Private stomatological hospital^AB^	40 (52.6)	36 (47.4)		
Private dental clinic^B^	80 (66.7)	40 (33.3)		

^*^P < 0.05.

^A, B, AB^Indicate significant differences at *P* < 0.05.

### 3.5. Dental professionals' exposure risk estimation and PPE use toward common dental procedures

The exposure risk score and PPE use score of each dental procedure are shown in Table 4. Among these procedures, ultrasonic scaling had the highest exposure risk estimation score, which was 4.28 ± 0.76. The Spearman correlation test found a highly statistically significant positive linear correlation between exposure risk scores and PPE use scores (*r* = 0.961, *P* < 0.01).

**Table 4:** Exposure risk estimation and PPE use of dental professionals toward common dental procedures.

**Dental procedures**	**Response**	**Exposure risk estimation**	**PPE use**
**Orthodontic treatment**			
Bonding of orthodontic attachments	262	2.68 ± 1.57	2.31 ± 0.80
Removal of orthodontic attachments	262	2.94 ± 1.63	2.37 ± 0.77
Orthodontic adhesive removal	261	3.43 ± 1.55	2.41 ± 0.77
Grinding of removable appliance	258	2.50 ± 1.55	2.28 ± 0.80
Micro-implant anchorage insertion	252	2.65 ± 1.63	2.24 ± 0.81
Fabrication of retainers	258	1.90 ± 1.53	2.16 ± 0.82
**Periodontal treatment**			
Ultrasonic scaling	161	4.28 ± 1.86	2.60 ± 0.76
Subgingival scaling	161	3.98 ± 1.27	2.57 ± 0.78
Air polishing	158	4.22 ± 1.28	2.59 ± 0.77
Periodontal flap surgery or gingival resection	145	3.43 ± 1.40	2.52 ± 0.81
**Filling or root canal treatment**			
Pulp opening	238	4.04 ± 1.31	2.56 ± 0.71
Infected tissue removal	238	3.55 ± 1.38	2.54 ± 0.73
Root canal filling	239	2.75 ± 1.41	2.47 ± 0.76
Grinding of filling materials	236	3.31 ± 1.64	2.52 ± 0.70

## 4. Discussion

### 4.1. Necessity, standards, and compliance of the PPE's proper use

The COVID-19 pandemic has caused a global crisis and captured the world's attention. During the outbreak, dental professionals were extremely concerned for their health and safety and that of their patients. Appropriately using PPE might considerably minimize the risk of viral transmission and infection. This survey provides insight into Chinese dental professionals' PPE use, with a special emphasis on COVID-19. The results showed that over half of (57.3%) Chinese dental professionals had unacceptable compliance with the proper use of PPE. This phenomenon was widely reported in studies of various countries. 53.2% of HCWs in an Egyptian survey admitted to not complying with the required usage of PPE (22). In Brazil, the compliance rate of HCWs with appropriate PPE use was observed to be 31.5% (23).

Surgical masks have been proven to prevent the transmission of human coronaviruses from symptomatic individuals and reduce the risk of COVID-19 infection (24, 25). Both particulate and bacterial filtration efficiency decline obviously with the increase of mask use time (26). According to protection standards for stomatological hospitals of China, dental professionals must keep wearing surgical masks during nursing, diagnosis, and treatment, and surgical masks are disposable and should be changed after 4 h of use, or immediately if the masks become moist or splattered (21). There were still 6.2% of dental professionals who could not always wear surgical masks, and 36.8% could not always change masks on time. It is worth noting that there are certain differences in definition and functionality between surgical masks in our study and respirators such as N95 and filtering masks. While both serve as PPE designed to reduce exposure, surgical masks are used to protect mucous membranes of the wearer's nose and mouth during activities that are likely to generate splashes or sprays, whereas respirators are used to prevent inhalation and protect the wearer from exposure to airborne hazards (27, 28).

SARS-CoV-2 can also be spread via contact with the conjunctiva due to the fact that infectious droplets can easily contaminate the conjunctival epithelium (29). A previous study has suggested that eye protection plays a role in the reduction of virus infection (25). Nevertheless, used dental goggles without effective and timely disinfection may become potential reservoirs for pathogens (30). Therefore, in order to protect the eyes from the hazards of aerosols and particulate matter generated in dental procedures, dental professionals are required to wear goggles or face shields and replace them in time when they are contaminated by blood, body fluids, secretions, etc. (21). Moreover, the used equipment should be cleaned, sterilized, and dried. Only 63.8% of the study participants always wore goggles or face shields, and only 45.6% always replaced them on time.

According to the definition by the Association for the Advancement of Medical Instrumentation (AAMI), the isolation gown is a piece of protective clothing used in patient isolation circumstances to prevent the spread of pathogens. Some studies have reported that using gowns plays a part in reducing the infection rate (31, 32), while the contaminated gown is considered a potential hazard for HCWs (33). The standard for the wear and change of isolation gowns is the same as surgical masks (21). Dental professionals in our study reported low compliance in isolation gown use, only 53.1% always wear isolation and only 36.72% always change on time.

In general, dental professionals reported relatively high compliance with surgical mask use, and relatively low compliance with goggles or face shields use and isolation gown use. This finding is consistent with Yang et al.'s study (34), and it could be attributed to Chinese intensive health education about mask wearing since the early outbreak. In addition, mask usage procedures are simpler, so it is easier for dental professionals to be compliant with the practice guidelines.

### 4.2. Personal barriers influencing compliance

Physical discomfort and glasses fogging were reported as the main barriers to the use of surgical masks. Most of our participants complained about the influence on operation and visual field caused by wearing goggles or face shields. One of the personal barriers to wearing isolation gowns is not easy for donning/doffing. These results are consistent with those of prior studies, which identified personal barriers, such as visual acuity, interference with operation, and comfort, as factors that influence PPE use compliance (35, 36). Because of this, the design and development of PPE need to take comfort and convenience into consideration while meeting the protection requirements. Besides, the necessity of wearing PPE, especially isolation gowns, in dental care settings should be emphasized because a considerable number of dental professionals have ignored it.

PPE's on-time change reported relatively low compliance. Most of our participants attribute it to the heavy workload. In addition, complex and time-consuming procedures of eye protection sterilization (34.0%) and gown changing (28.5%), which could further increase dental professionals' workload, were reported as important barriers to on-time change. HCWs' heavy workload was frequently attributed to the lack of human resources, particularly in developing countries (37). The desirable dentist-population ratio promulgated by WHO is 1:5,000 and it rises to 1:2,000 for developed countries. According to Chinese health statistics, the ratio of dentists to population is not more than 1:8,000 (20), which is much lower than the WHO's recommendation. We need to produce more dental graduates toward addressing the dental professional needs of the growing population.

### 4.3. Demographic factors influencing compliance

Our study found several demographic factors that significantly influenced dental professionals' compliance with the proper use of PPE, including age, years of practice, and type of medical institution. Dental professionals in the 31–40 age group and dental professionals with 11–15 practice years demonstrated significantly lower compliance compared to their counterparts. They are the same group of people because Chinese usually get their bachelor's degree at the age of 22 and 49.7% of our participants are graduates. This finding is consistent with a study conducted in Qatar, which revealed age and experience as significant predictors of PPE compliance (38). It might be explained by the fact that dental professionals at this age tend to work longer hours per week than those older and have higher job and family stress than those younger (39, 40). These factors could aggravate their job burnout and subsequently reduce their compliance.

Personal dental clinic professionals also showed lower compliance. A possible explanation for this might be that most private dental clinics in China are self-employed. Compared with public and private hospitals, these clinics usually are deficient in manpower, financial, and material resources and lack regulation and prior training on the proper use of PPE. In a survey of HCWs, knowledge, environmental context, and resources were found to be important factors affecting PPE use (34). During the pandemic, there is a significant increase in PPE demand and consumption. Inadequate supplies of PPE in medical institutions and a lack of knowledge make it difficult for HCWs to completely comply with the guidelines.

### 4.4. Exposure risk in dental procedures and dental professionals' estimation influencing compliance

Due to an individual's proximity and the production of aerosols in dental procedures, dental professionals are at high risk of COVID-19 transmission. Ultrasonic scaling, air polishing, and pulp opening were the top three dental procedures in the exposure risk estimation, and their mean scores were all over 4, 4.28 ± 1.86, 4.22 ± 1.28, and 4.04 ± 1.31, respectively. Dental professionals' estimation is consistent with previous experimental studies. In a study conducted by Choi, ultrasonic scaling produced a total aerosol volume of 4.18(±1.22) × 10^8^ μm^3^/m^3^ which was significantly higher (*P* < 0.001) than that of other dental procedures (41). In Logothetis' experiment, contamination caused by air polishing was detected 9 feet away in an operating room with 13 air changes/h (42). Zhu et al.'s experimental evaluation revealed that pulp opening produced aerosols with concentrations at least one order of magnitude above baseline (43). Public Health England has described dental procedures involving high-speed devices such as ultrasonic scalers and high-speed drills as creating an increased risk of respiratory infection transmission (44). Without exception, high-speed devices are continuously used during the above three procedures. At the same time, none of the procedures with a low exposure risk estimation (score below 3) need to use these devices.

Moreover, PPE use was found to be associated with exposure risk estimation. The finding is consistent with that of El-Sokkary et al. (22) who reported SARS-CoV-2 exposure risk as a crucial predictor for compliance with the proper use of PPE (22). The results showed that dental professionals could recognize the risks of using high-speed devices and strengthen their preventive practice. Hospital managers can strengthen the training of dental professionals on risk perception to improve PPE use.

### 4.5. Strengths and limitations

To the best of our knowledge, this seems to be the first nationwide study in China to assess dental professionals' PPE use during the COVID-19 pandemic. Our study covered a wide range of Chinese dental professionals, and the participants' demographic characteristics corresponded with the national statistics. We assessed the status of PPE use, analyzed its influencing factors, and provided dental participants with some practical recommendations.

Several limitations existed in our study. Firstly, there was possible selection bias among study participants. This survey was conducted online, and only 20 provinces were involved, so the findings may not represent all Chinese dental professionals. Secondly, all data for analysis was gathered from self-administered questionnaires. There may have been dishonesty among certain participants. However, we are confident that the survey's anonymity allowed the participants to freely describe their behavior and increased the study's credibility.

## 5. Conclusions

Chinese dental professionals who participated in this survey revealed low compliance with the proper use of PPE during the COVID-19 pandemic, with 57.3% reporting unacceptable compliance. The compliance of eye protection (goggles or face shields) use and isolation gown use was far from satisfactory, as well as the on-time change implementation rate. Multifarious personal barriers were reported, the most common of which were visual barriers, physical discomfort, complex procedures, and heavy workload. Dental professionals' PPE use practice was significantly correlated with age, years of practice, type of medical institution, and estimation of exposure risk.

Greater efforts on all sides are needed to improve dental professionals' PPE use compliance in response to the COVID-19 pandemic or other possible future pandemics. It is crucial for dental institutions to have an adequate supply of PPE and sufficient human resources. Comprehensive training interventions on PPE use practices are also necessary. These interventions should include education on selecting the right PPE, proper procedures of donning, doffing, and disposal, as well as emphasizing the importance of perceiving exposure risks. The design and development of PPE need to take comfort and convenience into consideration to improve use compliance. For dental professionals, especially those in the 31–40 age group, with 11–15 experience years, or working in private dental clinics, it is vital to constantly learn and comply with the best practice and recommended guidelines of PPE.

## Data availability statement

The raw data supporting the conclusions of this article will be made available by the authors, without undue reservation.

## Ethics statement

The studies involving human participants were reviewed and approved by Ethics Committee of Zhongda Hospital of Southeast University. Written informed consent for participation was not required for this study in accordance with the national legislation and the institutional requirements.

## Author contributions

QW, LH, and XZ contributed to the design and conception of the study. QW analyzed and interpreted data and wrote the first draft of the article. LH collected and organized data, as well as contributed substantially to writing the manuscript. XY and SY contributed to the design of the questionnaire. XZ involved in supervision, reviewing, and editing. All authors contributed to manuscript revision, read, and approved the submitted version.
